# Elementary Obstetrics

**Published:** 1900-12

**Authors:** W. E. A. Wyman, T. F. Moyle

**Affiliations:** Milwaukee, Wis.; Waterford, Wis.


### ELEMENTARY OBSTETRICS.

By W. E. A. Wyman, M.D.V., V.S.,
MILWAUKEE, WIS.,

AND

T. F. Moyle, V.S.,
WATERFORD, WIS.

The veterinarian who selects the country as his field of labor will of necessity be called upon to do more or less obstetrical work.

Those who succeed in establishing a reputation as obstetricians do so, in the vast majority of cases, at the expense of their clients and patients. It is not our desire to dissect obstetrical teaching as the colleges furnished it and as it is still offered by some. How many collegiate obstetricians of to-day do not have a kindly feeling for the farmer who helped them make their first case of dystocia a success? Charity begins at home, and practical obstetrics should begin at college. But does it? Since that important part of the young veterinarian's clinical training is—to put it mildly—neglected, the conscientious student necessarily looked for other channels of instruction, namely, literature on the subject of practical obstetrics. All those acquainted with other languages but English did find treatises dealing with that subject to some extent, but most of them of antediluvian date, representing able but antique views, quite incompatible with the present times. Fleming's *Obstetrics*, being a compilation of what we discussed a moment ago, might be classed with them. While intensely thankful for the contents of such a work, representing a great amount of labor, nevertheless it is inadequate, needing revisions and additions as much as most obstetrical chairs from which many of us were supposed to gather such common and original facts as to render us fit to at least compete with the farmer obstetrician. These few remarks represent truth, and the progressive spirit which begins to exhibit itself among the colleges will no doubt find means to elevate the department of obstetrics to the proper standard.

The following points are those which in the course of time were gathered in an extensive obstetrical practice, and are recommended as tried facts, with our best wishes.

It cannot be denied that a long arm with a slender and strong hand is likely to be of more service than the opposite anatomical arrangement. Nevertheless, the obstetrician equipped with the latter need not despair.

During warm weather it matters but little what he wears, but in the winter some precautions are necessary to prevent the contraction of a more or less serious cold. Do not remove any underwear for obvious reasons; put over it either a heavy armless woollen shirt or cardigan jacket, and over this an operating apron which is split a little above the knees so that each half can be tied around the corresponding leg a little below the knee. Next order a clean pail of hot water, put into it a proper amount of Parke, Davis & Co.'s germicidal soap, and as arms and hands get cold and soiled bathe them in this hot water containing mercuric iodide. The results are: Disinfection and no cold, two vital points to the much-exposed rural practitioner. Also get some clean towels when ordering the water. It is well to emphasize the fact that you want a clean pail and a clean towel, as nine farmers out of ten would bring a swill pail and some old dish-rag—rather incompatible with asepsis.

Being taken care of yourself, secure a place which is adequate for the occasion; in other words, make room for yourself and patient. If the stall slopes much and has a gutter at the end, have the owner bring in straw, or, better yet, cornstalks, and fill the gutter so that the cow cannot slip and possibly fall. Fix the head of the cow with a five-ring halter, and, if possible, call for two able-bodied men. In giving orders issue them pleasantly but firmly. The farmer, with his tendency to become a jack of all trades, is very likely to disagree with you, and offer amendments. The more serious the case, the less will he encourage you, and since a great many may have exhausted their own and neighbors' skill in trying to deliver the cow or mare, as the case may be, the owner as well as all bystanders—sometimes quite a crowd—could hardly see how anybody else could succeed after they failed. Anything you do or say is acutely observed, and only too often poorly interpreted by those about.

Questions put to you are often overheard, as both the place and person, as a rule, are unfit for arguments. Exhibit no undue haste in word or deed, but impress them with the fact that you superintend the whole thing, and, above all, keep the helpers busy, especially those who constantly instruct you.

We will suppose the animal to be standing up and properly fastened with a halter; tail, vagina, and perineum have been cleansed with germicidal soap and water, your hands disinfected, nails trimmed, etc. The question arises, Is she ready to give birth? Has she gone her full term or not? In the case of the cow the farmer does not usually know the day or month when she is due to calve, on account of the ruinous practice of letting the bull run with the herd. It is quite important to settle that point, as false pains may have alarmed the owner and caused him to send for the surgeon. On vaginal exploration in false pains the mucus covering the palmæ plicatæ like a stopper still adheres firmly, and the os uteri is closed, the pains are short and sharp, the animal appears hyperæsthetic, kicks, and switches the tail. In such a case leave her alone, and, since most farmers would be greatly dissatisfied unless she gets some medicine, give her a bromide of potassium treatment. On the other hand, when the cow on your arrival is past false pains, and entered the period of true labor pains—dolores—you find the external os uteri more or less dilated, the otherwise sticky mucus of the palmæ plicatæ has become detached, and part of it is lying in the vagina or possibly hanging from it. Palpate the pelvic canal carefully, both bony frame and soft parts, to see whether the dimensions permit the passage of the young. In the cow it is well to empty the udder, provided it is full and strutting. Many a cow rendered uneasy by the distended udder will become quiet as soon as she is milked, and from that moment attends to her business nicely. Quite often objections are raised right here, but insist upon the milking, as it is your duty to protect the owner against his own ignorance and superstitions, at least in such a case. Sometimes it occurs that the cow will not rise, and obstinately refuses to respond to the ordinary means employed to make her get up. In such a case a good yelping dog taken to her head will do wonders. When a mare is down and unable to rise do not waste any time trying to induce her to rise. As regards the cow when in a recumbent position, endeavor to keep her on the right abdominal side as much as possible, as continuous pressure upon the rumen when lying on the left side results in paresis of that organ, which means tympanites and discomfort. Should the fœtal membranes have been ruptured and the genital passage is dry, the uterus, as the result of aspiration, will adapt itself closely to the fœtus, rendering manipulations difficult. In such a case inject water at body temperature into the uterus. In the mare call for five pounds of lard and pass it in the shape of a ball as far back into the genital passage as possible.

Some cows, the genitals of which are hyperæsthetic, jump around, throw themselves, etc., rendering any work impossible. Here order two boxes as high as the abdomen, and one twelve-inch board; pass the board under the belly right in front of the udder, resting the two ends of the board on the boxes. The cow now goes down in front, while the elevated posterior extremity is in nice shape for an examination or delivery. When down and unable to rise, cow or mare, put the hobbles on them, elevate them behind by means of straw or cornstalks. On the whole, it is a good rule to work with your patient elevated behind, as it helps to overcome a great many obstacles. Inability to make any progress in securing a leg or head, for instance, while the mother rests on the right side, should induce you to put her into a dorsal position, and if that does not work try her on the left side, etc. "Finish whatever you undertake—persistence is a fundamental factor in obstetrics." Breech presentations will tax your skill, strength, and patience, and of the latter always take an extra set along. If you cannot lasso the posterior extremities of the young in a breech presentation, fasten a strong rope or chain to a beam overhead and raise the hindquarters of the female. While repelling the young in this position, it standing, so to speak, on its head, be sure that the whole fœtus moves along, or the head, when caught anywhere about the uterus, will rupture it. In such a case the mare is best chloroformed. When cording any part of the fœtus, convince yourself that the cord encloses only parts of the fœtus and not a portion of the fœtal membrane, as subsequent traction might sever some cotyledons, exposing the animal to a serious hemorrhage. In the employment of traction while delivering the young, remember the line of guidance: as the fœtus enters the pelvic canal pull in the direction of the sacrum—upward. As it passes through the canal exert downward traction, and as it leaves the maternal passage, again pull upward, but pull only during the exhibition of labor pains. When repelling a fœtus the frequent pains often throw the young toward you again and again, interfering with the regular course materially. In such a case hold the fœtus steady during one or two throws, and in the majority of cases you may now repel without experiencing much resistance from the mother. On an average, the power exerted by three men ought to be the limit of traction employed in extracting a fœtus. Of course, there are instances where greater power by far may be safely employed, for instance, in the delivery of an emphysematous calf.

After the young is born let the mother lick it, or, if circumstances prevent, have it rubbed dry, and in the colt give surgical attention to the umbilical cord—apply some desiccating antiseptic, a mixture of tannic acid and acetanilid being very useful.

To prevent excessive after-pains and possibly eversions of the uterus, elevate the mother behind. Cleanse all parts that became soiled, and unless the calf is allowed to suck the colostrum have her milked. Feed lightly at first, and mothers of a lymphatic temperament might be given a saline laxative.

Remove the placenta in the cow forty-eight hours after delivery, while immediate removal is essential in the mare, and to the one removing a mare's after-birth for the first time we suggest care and patience; loosen it by lifting the membrane from its attachment; sometimes an hour may be required to remove it *in toto*. When a piece is left in the uterus the mare soon begins to strain violently, and by the time you have reached your office, congratulating yourself upon the success of the task, the farmer will be after you with a club.

What obstetrical instruments are required? Certainly not those which are ordinarily offered for sale. If you have a set, take them along. Spread them out and utilize those bright, impractically-shaped, office-constructed things to hypnotize the audience, or, better yet, hide them, as even your farmer customer, with his practical turn of mind, might point out their deficiencies. Have one blunt and one sharp hook, one repeller, no less than four feet long; one set of pulleys carrying a nine-sixteenths of an inch rope (thirty feet); guiding ropes (window-cords, cotton ropes), you need four, each not less than four feet long, and each with a stationary eye, so that the rope may pass through with ease, as your fingers get tired lassoing legs, neck, head, etc.

Drugs needed are: antiseptics, such as creolin, formalin, alum, and Parke, Davis & Co.'s germicidal soap (the latter we prefer). Alcohol as a stimulant; strychnine as a nerve tonic; spiritus ammoniæ aromaticus and ol. terebinthinæ as stimulants and anti-fermentatives; and, finally, fluid extract of secale cornutum when the uterus is exhausted.

